# Clindamycin susceptibility and virulence characterization of *Listeria monocytogenes* strains isolated from meat and meat-processing environments

**DOI:** 10.3389/fmicb.2026.1833569

**Published:** 2026-05-21

**Authors:** Aida Pérez-Baltar, Bibiana Hernández-Gelabert, Pilar López, David Pérez-Boto, Juan Luis Arqués, Joaquín Venancio Martínez-Suárez, Raquel Montiel

**Affiliations:** Departamento de Tecnología de Alimentos, Instituto Nacional de Investigación y Tecnología Agraria y Alimentaria-Consejo Superior de Investigaciones Científicas (INIA-CSIC), Madrid, Spain

**Keywords:** antimicrobial resistance, clindamycin, genotyping, *Listeria monocytogenes*, resistance genes, virulence

## Abstract

*Listeria monocytogenes* is a major foodborne pathogen widely distributed in food-processing environments and is considered intrinsically resistant to clindamycin. The aim of this study was to investigate the relationship between clindamycin susceptibility and virulence in *Listeria monocytogenes* strains isolated from meat and meat-processing environments belonging to different clonal complexes (CCs). Clindamycin susceptibility in *L. monocytogenes* strains was phenotypically and genotypically characterized by disk diffusion method, minimum inhibitory concentration (MIC) determination, and detection of resistance-associated genes. Whole-genome sequencing was used to compare strains with different susceptibility profiles and to explore the relationship between clindamycin resistance and virulence factors. Among 62 strains analyzed, 22.6% were phenotypically susceptible, showing MIC values between 0.25 and 0.5 μg/mL. The clindamycin resistance gene *vgaG* was detected in both resistant and susceptible strains, with a potentially deleterious mutation identified in susceptible CC155 strains. All genomes harbored the additional intrinsic antibiotic resistance genes *fosX, norB, mprF*, and *sul*. The clindamycin susceptible strains also harbored truncated or impaired virulence genes, including *prfA, inlA, inlB, actA,* and *plcA*, which correlated with the absence of phosphatidylinositol-specific phospholipase C activity on CHROMagar™ *Listeria*. Premature stop codons were detected in *inlA* in strains belonging to CC31 and CC155, as well as a truncated variant of *prfA* in CC31 strains, both alterations associated with hypovirulent phenotypes. Overall, these findings provide new insights into the interplay between antimicrobial resistance and virulence in *L. monocytogenes*, and highlight the relevance of genomic surveillance in food-processing environments.

## 1 Introduction

*Listeria monocytogenes* is a Gram-positive bacterium classified into four lineages (I–IV) and 14 serotypes (Varsaki et al., 2022), being the only species among the 29 included in the genus *Listeria* that is recognized as a human pathogen according to the List of Prokaryotic names with Standing in Nomenclature (LPSN) (Bouznada et al., 2024). This bacterium causes listeriosis, a foodborne infection primarily transmitted through the consumption of contaminated food. Due to its wide environmental distribution, ability to form biofilms on a variety of surfaces, and tolerance to suboptimal conditions, *L. monocytogenes* is considered one of the most concerning pathogenic bacteria in the food industry.

Listeriosis is considered the most severe foodborne disease in terms of hospitalization (more than 97% of cases) and has the highest mortality rate in the European Union, accounting for 15.6% in 2024 (EFSA and ECDC, 2025). *L. monocytogenes* virulence is mediated by a set of genes that regulate its intracellular life cycle, including cell invasion (*inlA*, *inlB*), vacuolar escape (*hly*, *plcA*, *plcB*, *mpl*), intracellular replication (*hpt*), and cell-to-cell spread (*actA*). The expression of these genes is primarily controlled by PrfA, a key regulatory factor for the differential expression of virulence genes within the *L. monocytogenes* pathogenicity island 1 (LIPI-1) and additional chromosomal loci such as the *inlAB* operon. PrfA, whose expression is positively regulated by the Sigma factor (encoded by *sigB*), plays a central role in coordinating its pathogenicity (Vázquez-Boland et al., 2001; Koopmans et al., 2023).

One of the major public health challenges of the 21st century is the emergence and spread of antibiotic-resistant bacteria (WHO, 2022). Research on the antibiotic resistance of *L. monocytogenes* has produced contradictory results, partly due to the lack of standardization in antimicrobial susceptibility testing methods (Montiel et al., 2025). Using the standardized method proposed by the European Committee on Antimicrobial Susceptibility Testing (EUCAST, 2025), *L. monocytogenes* shows a high rate of susceptibility to antibiotics commonly employed in the treatment of listeriosis, (Baquero et al., 2020; Montiel et al., 2025; Moura et al., 2024). Nonetheless, the pathogen can acquire antibiotic resistance genes from other organisms through plasmids, prophages, or mobile genetic elements such as transposons (Baquero et al., 2020; Moura et al., 2024). Moreover, the exposure to sub-inhibitory concentrations of disinfectants may induce bacterial resistance and promote cross-resistance to antibiotics (Walczak et al., 2026). Consequently, monitoring the resistance of this pathogenic bacterium is crucial in specific settings such as the food industry and hospitals, where selective pressure is higher due to the frequent and widespread use of disinfectants and antibiotics (Montiel et al., 2025).

Clindamycin, a lincosamide-class antibiotic, is classified as a highly important antimicrobial for use in both human and veterinary medicine (WHO, 2024). *L. monocytogenes* exhibits natural or intrinsic resistance to clindamycin (CA-SFM and EUCAST, 2024), mediated by the expression of the constitutive core gene *vgaG* (*lmo0919*), which encodes an ABC-F type ribosomal protection protein that competes for the clindamycin binding site (Dar et al., 2016). Clindamycin resistance may also result from the presence of plasmid-borne resistance genes, such as *lnuA, lnuB,* and *lnuG* (Chmielowska et al., 2021; Rodrigues et al., 2024), which encode nucleotidyltransferases that catalyze clindamycin adenylation, thereby inactivating the antibiotic. However, this form of acquired resistance is comparatively rare (Moura et al., 2024).

Although *L. monocytogenes* is intrinsically resistant to clindamycin, a substantial number of phenotypically susceptible isolates have been registered (Moura et al., 2024; Díaz-Martínez et al., 2025). The lack of correlation observed between phenotypic and genotypic resistance profiles in clinical and food-associated strains complicates the interpretation of susceptibility testing results and hinders the development of effective control and treatment strategies. This controversy makes clindamycin a suitable model for investigating the relationship between intrinsic antibiotic resistance determinants and virulence-associated traits. Such information is particularly relevant for strains isolated from the food industry, a high-risk environment for the selection and dissemination of adapted variants. Accordingly, the aim of this study was to assess the relationship between phenotypic and genotypic clindamycin resistance profiles and the presence of virulence-associated genes in *L. monocytogenes* strains from meat-processing environments, to improve the understanding of antimicrobial resistance dynamics in this context.

## 2 Materials and methods

### 2.1 *Listeria monocytogenes* strains

A total of 62 *L. monocytogenes* strains from the culture collection of INIA-CSIC (Instituto Nacional de Investigación y Tecnología Agraria y Alimentaria, Madrid, Spain) were included in this study (Supplementary Table S1). The strains had been isolated from different poultry or pork production companies in Spain and have been previously described in independent studies (López et al., 2007, 2008, 2013; Ortiz et al., 2010; Pérez-Baltar et al., 2021). All strains were previously subtyped by PCR serogrouping (Doumith et al., 2004), PFGE (Halpin et al., 2010) and MLST (Ragon et al., 2008). This information is compiled in the studies by Ortiz et al. (2010, 2014), López-Alonso et al. (2020) and Pérez-Baltar et al. (2021). Strains were stored at −80 °C in Brain Heart Infusion broth (BHI, Biolife S.r.l., Milan, Italy) supplemented with 15% glycerol. Prior to use in experiments, strains were streaked onto Tryptic Soy Yeast Extract Agar (TSYEA, Sigma-Aldrich Chemical Co., MO, United States) and incubated at 37 °C for 24 h.

### 2.2 Phenotypic characterization

#### 2.2.1 Growth in CHROMagar-*Listeria*

The growth and halo formation of 62 *L. monocytogenes* strains were assessed at 37 °C on CHROMagar™ *Listeria* (CH-L, Scharlab S. L., Barcelona, Spain) and compared to the reference strain *L. monocytogenes* ATCC BAA-679 (EGD-e) (Glaser et al., 2001), whose halo size was designated as “++.” Halos of larger diameter were classified as “+++,” equal size as “++,” and smaller halos as “+.” Absence of halo was recorded as “−.”

#### 2.2.2 Clindamycin susceptibility analysis: disk diffusion method and minimum inhibitory concentration

The 62 *L. monocytogenes* strains were analyzed to determinate the antibiotic susceptibility on Müller-Hinton (MH) agar supplemented with 5% defibrinated horse blood and 20 mg/L β-NAD (Müeller-Hinton Fastidious, MH-F, Becton Dickinson GmbH, Heidelberg, Germany) according to the standard disk diffusion method recommended by the EUCAST (2025). Clindamycin (2 μg/disk) obtained from Oxoid (Thermo Fisher Scientific, MA, United States) was used. Plates were incubated at 35 °C for 18 h in an anaerobic jar with 5% CO₂. The assay was performed in duplicate, with each replicate including duplicate samples. Inhibition zone diameters were measured on open plates under reflected light and interpreted according to the criteria established by Moura et al. (2024), as this antibiotic is not included in the standardized method of EUCAST. *L. monocytogenes* ATCC BAA-679 and *Staphylococcus aureus* ATCC 29213 were used as control strains.

Based on the results of disk diffusion assay, 16 *L. monocytogenes* strains were selected to determine the minimum inhibitory concentration (MIC), 14 strains susceptible to clindamycin and two resistant strains. *L. monocytogenes* strain ATCC BAA-679 was used as control. The MIC was determined through the agar dilution method (CLSI, 2024) on MH agar plates containing two-fold serial dilutions of clindamycin, and the plates were incubated at 35 °C for 24 h. The assay was performed in triplicate. Growth of the strains was evaluated on open plates under reflected light. The MIC was defined as the lowest concentration of clindamycin that inhibits the visible growth of *L. monocytogenes* strains, expressed in μg/mL.

### 2.3 Genotypic characterization

#### 2.3.1 *In vitro* detection of clindamycin resistance genes in *Listeria monocytogenes*

The strains were screened by PCR for the presence of clindamycin resistance genes, specifically, *lnuB, lnuG,* and *vgaG* genes. A rapid extraction of total DNA was performed from pure *L. monocytogenes* cultures in TSYEA plates incubated at 37 °C for 24 h. Several colonies were resuspended in a mixture of 0.25% sodium dodecyl sulfate (SDS, Panreac Química SLU, Barcelona, Spain) and 0.05 M sodium hydroxide (NaOH, Panreac). Lysis was performed by heat and, after centrifugation, the supernatants containing DNA were recovered and stored at −20 °C until use. Specific primers for partial amplification of *lnuB, lnuG,* and *vgaG* genes were designed (Table 1). Each 20 μL-PCR reaction contained 200 nM of each primer and 2 μL of extracted DNA from *L. monocytogenes* isolates. The PCR amplification conditions were as follows: an initial cycle at 95 °C for 5 min; 35 amplification cycles, each consisting of 95 °C for 30 s, 52 °C for 30 s, and 72 °C for 2 min; and a final incubation at 72 °C for 7 min.

**Table 1:** PCR and sequencing primers used in this study.

Locus	Putative function of gene		Sequence (5′ → 3′)	Amplicon size (bp)
Antimicrobial resistance genes to lincosamides				
*lnuB*	Lincosamide nucleotidyltransferase		F: TCGTTTACCAAAGGAGAAGGT	328
			R: TTGGTCTTGCACCACTTATCT	
*lnuG*	Lincosamide nucleotidyltransferase		F: CGAAATTCCATAACGAGAAGC	702
			R: GGCAAGGGTTAAGGAACTTG	
*vgaG*	ABC-F type ribosomal protection protein		F: TAGCGTTCAAACCAAGCAAG	466
			R: TTCGAGCATGGCTTTTTCTG	
Sequencing primers				
*vgaG*			F: CGGCTTATAAAGGTTGAAGTA	1,918
			R: AACTATTTGATTCTAAGCCTATCT	
*vgaG* internal sequencing primers			R1: TTTTTGTTCTAAATACGCGTG	
			F2: CATCAAACAACTTTCCACG	
			R2: TACTTGCTTGGTTTGAACG	
			F3: CTATTAAAGCAGGGGATAAAGT	

Subsequently, the antibiotic resistance genes detected by PCR were sequenced and analyzed to determine possible variations among strains. Amplified fragments were purified using the mi-PCR Purification Kit (Metabion International AG, Planegg, Germany), following the manufacturer’s instructions. After purification, fragment quality and concentration were assessed with a NanoPhotometer Implen N60 (Implen GmbH, Munich, Germany). Sequencing was performed by the Sanger sequencing service of STAB Vida (Caparica, Portugal). The sequencing products of the *vgaG* gene were assembled and analyzed using BIOEDIT Sequence Alignment Editor 7.0.9 (Ibis Biosciences, Carlsbad, CA, United States), and compared with the allelic profiles available in the Institut Pasteur Bacterial Isolate Genome Sequence Database (BIGSdb-Lm v1.52.0). The nucleotide sequences obtained were translated into their corresponding amino acid sequences using the ExPASy translate tool (Swiss Institute of Bioinformatics, Laussane, Switzerland) to identify potential variations that could affect protein functionality. SIFT (Sorting Intolerant From Tolerant), a tool for predicting the functional impact of amino acid substitutions, was then used to evaluate the potential effect of the observed variants at a 95% confidence interval (Sim et al., 2012).

#### 2.3.2 *In silico* molecular characterization using whole-genome sequencing

Based on the results of phenotypic analysis, 10 *L. monocytogenes* strains were selected for molecular characterization through whole-genome sequencing (WGS). These included eight clindamycin susceptible strains, belonging to clonal complexes (CCs) 31 (A7, A13, C009, R4, and S1) and 155 (P8, P9, and R1), as well as two clindamycin resistant strains, one from CC155 (C007) and the other from CC121 (A001).

##### 2.3.2.1 WGS, assembly and annotation

The strains were grown on TSYEA plates at 37 °C for 24 h. A single colony was transferred to BHI broth and grown at 37 °C overnight. Genomic DNA from the strains was purified using the Microbial gDNA isolation kit (NZYtech, Lisbon, Portugal) following the manufacturer’s instructions. Sequencing was performed by the WGS service of STAB Vida. For library construction, genomic DNA was mechanically fragmented (sonicated), end-repaired and A-tailed. Sequencing adapters and Indexes were ligated using the KAPA HyperPrep workflow (Roche, Basel, Switzerland). Libraries were purified and size-selected using magnetic beads, quantified, evaluated, normalized, and finally sequenced by the Illumina NovaSeq 6000 platform (Illumina, San Diego, CA, United States), to obtain 150-bp paired-end sequencing reads. FastQC software (v0.12.1) was used to analyze the quality control of the obtained individual reads (Andrews, 2025) and more than 85% of bases had a high quality, greater to Q30. Reads were assembled using the software CLC Genomics Workbench v12.0.3 (QIAGEN, Hilden, Germany), which employs an algorithm based on de Bruijn graphs (Compeau et al., 2011), with a length fraction of 0.80, similarity fraction of 0.80 and minimum contig size of 500 bp as parameters. Genome assemblies quality was evaluated with QUAST 5.2.0 (Mikheenko et al., 2018). Annotation of whole genome assemblies was carried out using Prokka v1.14.6 (Seemann, 2014) from Galaxy website, and Rapid Annotation using Subsystem Technology Toolkit (RASTtk) from the Bacterial and Viral Bioinformatics Resource Center (BV-BRC) (Brettin et al., 2015).

##### 2.3.2.2 WGS analysis

Comparative analysis of the coding DNA sequences (CDS) among the *L. monocytogenes* strains, comprising 10 strains from meat-processing environment and the reference strain ATCC BAA-679, was performed using the Roary pangenome pipeline (Page et al., 2015), available on the Galaxy platform, allowing identification of core, accessory, and unique genes across all 11 genomes. The whole-genome alignment was generated using the BV-BRC Genome Alignment Service and visualized with the PathoSystems Resource Integration Center (PATRIC) Mauve Viewer to examine large-scale rearrangements and conserved genomic regions. Phylogenetic analysis was carried out using the Bacterial Phylogenetic Tree Service available on the BV-BRC platform, which selects single-copy conserved genes, aligns them using MAFFT (Multiple Alignment using Fast Fourier Transform), and infers evolutionary relationships through a Maximum Likelihood tree generated with RAxML (Randomized Axelerated Maximum Likelihood).

##### 2.3.2.3 Genetic determinants of virulence and antibiotic resistance

The presence of genes associated with virulence and resistance to antibiotics was screened using several tools. First, *L. monocytogenes* genomes were analyzed using the BIGSdb-Lm database. Additionally, the ABRIcate tool (v1.0.1) on the Galaxy Europe platform was employed, and analyses were performed using the Virulence Factor Database (VFDB), the Comprehensive Antibiotic Resistance Database (CARD) and Resfinder. If these tools failed to identify the target genes, Basic Local Alignment Search Tool (BLAST), v2.17.0 from the National Center for Biotechnology Information (NCBI) was then used. Allelic differences in the genes of interest were compared with the allelic profiles of the reference strain *L. monocytogenes* ATCC BAA-679 from BIGSdb-Lm. Gene sequences were translated and aligned as described in section 2.3.1 to assess potential differences in the amino acid sequences of the corresponding proteins. Possible losses of protein functionality were evaluated using SIFT. Likewise, the tools PlasmidFinder (v2.1) (Carattoli et al., 2014) and MobileElementsFinder (v1.0.2) (Johansson et al., 2021) were used to analyze the potential presence of plasmids and mobile genetic elements, respectively, that could carry antimicrobial resistance genes. Only those with an identity greater than 95% and a coverage higher than 60% were considered.

##### 2.3.2.4 Data availability

The Whole Genome Shotgun project has been deposited at DDBJ/ENA/GenBank under BioProject PRJNA1435101, with the individual accession numbers: A001 (JBWDFT000000000), C007 (JBWDFU000000000), R1 (JBWDFV000000000), P9 (JBWDFW000000000), P8 (JBWDFX000000000), C009 (JBWDFY000000000) and R4 (JBWDFZ000000000). Three strains had previously been sequenced by López-Alonso et al. (2015, 2019) with the following GenBank accession numbers: A7 (RXOS00000000), A13 (RXOR00000000) and S1 (JWHI00000000), although genes associated with antibiotic resistance or virulence were not analyzed in those studies.

### 2.4 Data analysis

Statistical analyses were carried out using SPSS Statistics 22.0 software (IBM Corp., Armonk, NY, United States). A correlation analysis was performed to assess associations between the variables of interest by determining the Phi coefficient (*ϕ*). Significant differences (*α* = 0.05) in correlation analysis were studied by Fisher’s Exact Test. The non-parametric Kruskal-Wallis test was carried out to evaluate differences (*α* = 0.05) in the distribution between the variables across groups.

## 3 Results and discussion

### 3.1 Phenotypic characterization

#### 3.1.1 Growth in CHROMagar-*Listeria*

**Figure 1:**
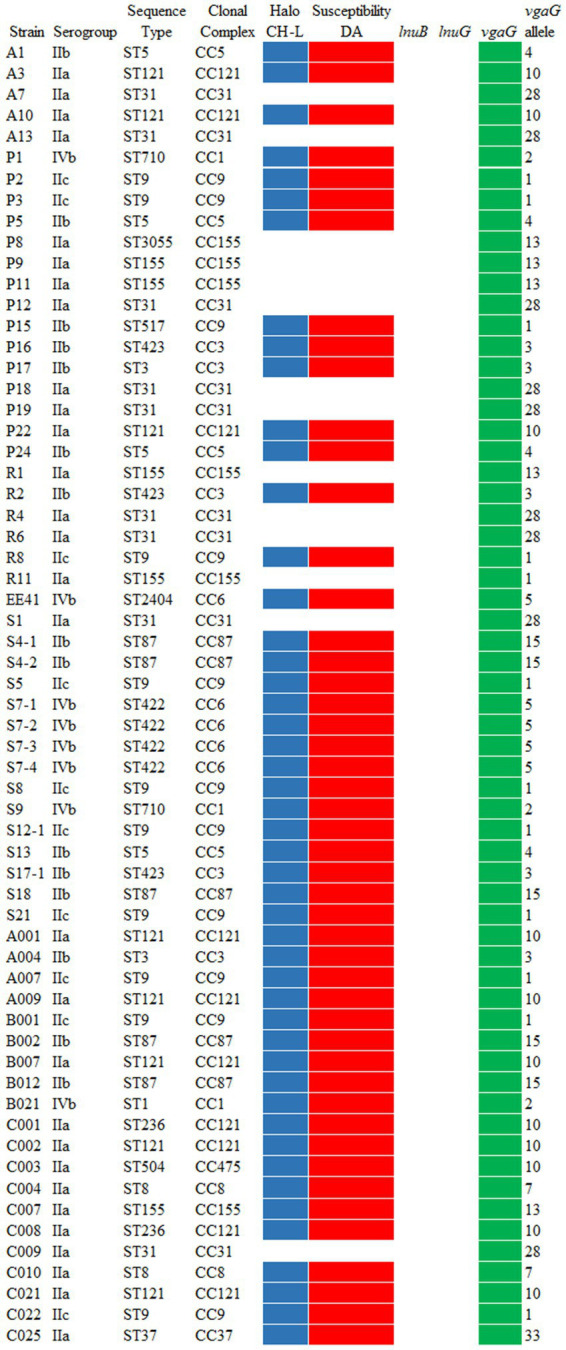
Characterization of 62 *L. monocytogenes* strains isolated from meat-processing industries. Blue colored boxes indicate halo formation in CH-L medium. Red boxes indicate clindamycin resistance determined by the EUCAST disk diffusion method. Green boxes indicate the presence of clindamycin resistance genes. CH-L, CHROMagar *Listeria* medium; DA, clindamycin.

Growth and halo formation in CH-L medium were studied in 62 strains of *L. monocytogenes*. In total, 77.4% of the strains (48/62) produced typical blue colonies surrounded by a white halo, with a halo size similar to that of the reference strain ATCC BAA-679 (“++”). In contrast, 22.6% of the strains (14/62) formed typical colonies but without halo (“–”) (Figure 1; Supplementary Table S2). Halo-producing strains were assigned to CCs 1, 3, 5, 6, 8, 9, 37, 87, 121, 155, and 475, whereas non-halo forming strains belonged to CCs 31 and 155. All strains within a CC showed consistent behavior, except for CC155. Within this CC, strains P8, P9, P11, R1, and R11 lacked halo formation, while strain C007 produced a halo comparable to the reference strain.

CH-L is a chromogenic, selective, and differential medium containing L-α-phosphatidylinositol, hydrolyzed by phosphatidylinositol-specific phospholipase C (PI-PLC) of *L. monocytogenes* to produce a white precipitate around the colony, and a chromogenic substrate (5-bromo-4-chloro-3-indoxyl-β-d-glucopyranoside) cleaved by β-d-glucosidase, resulting in blue colonies (Reissbrodt, 2004). Consequently, CH-L medium allows the identification of *L. monocytogenes* colonies, which display blue growth surrounded by a white precipitate halo, as observed in many of the strains analyzed. The absence of halo formation may be associated with alterations in virulence-related genes, such as *plcA*, which encodes PI-PLC, or *prfA* which regulates virulence genes within LIPI-1, including *plcA* (Koopmans et al., 2023; de las Heras et al., 2011). Strains belonging to CC31 and CC155 have been described as hypovirulent due to mutations in virulence genes including *prfA* and *inlA,* and have been associated with reduce invasion capacity and non-hemolytic phenotypes (Roche et al., 2012; Maury et al., 2017). In this study, the absence of halo formation in CH-L medium was observed exclusively in strains from these CCs, suggesting a link with virulence gene variability. Therefore, CH-L medium, beyond identification purposes, may provide preliminary indications of virulence-related traits.

#### 3.1.2 Clindamycin susceptibility of *Listeria monocytogenes*

**Figure 2:**
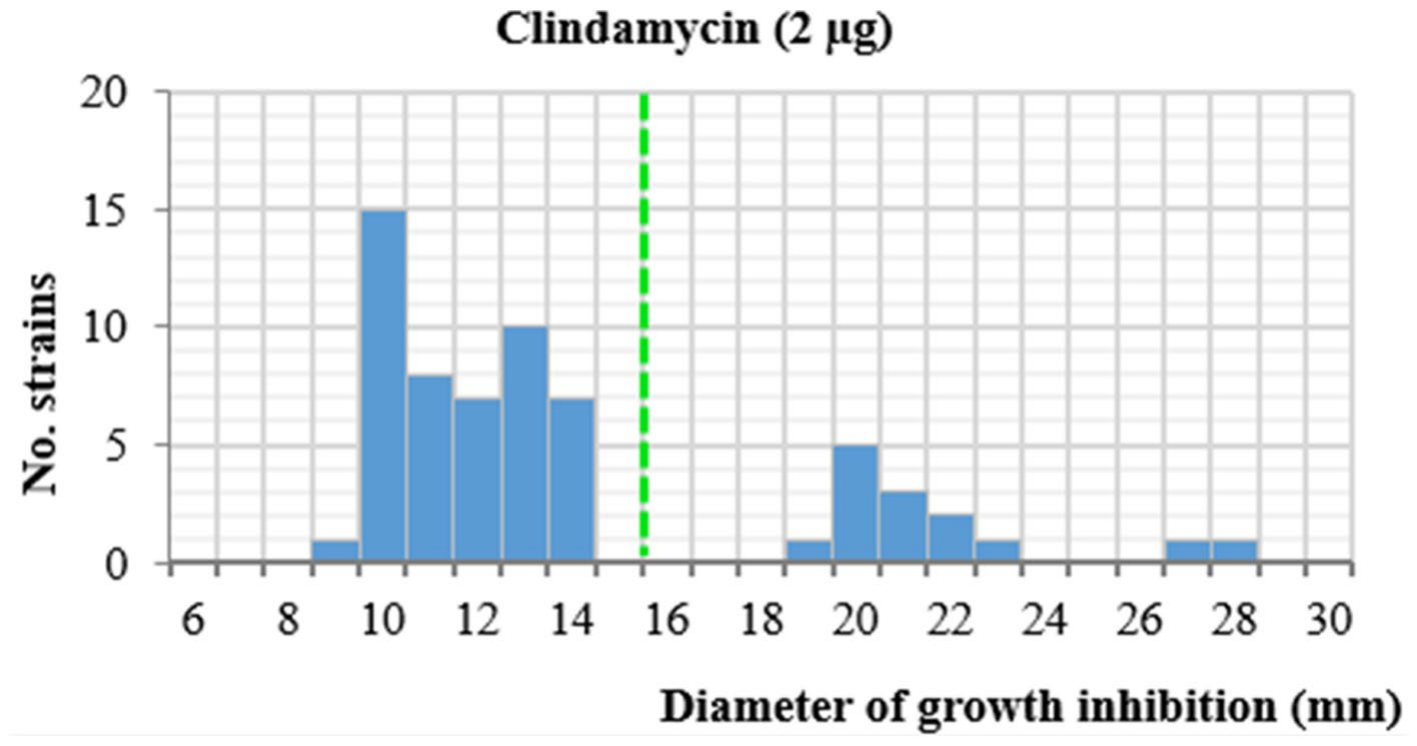
Distribution of inhibition diameters (halos) for 62 strains of *L. monocytogenes* against clindamycin. Dotted lines correspond to resistance diameter breakpoint.

The susceptibility of 62 *L. monocytogenes* strains to clindamycin, a lincosamide-class antibiotic commonly used against Gram-positive bacteria infections (Wiśniewski et al., 2022), was evaluated following the EUCAST disk diffusion protocol. This method only provides breakpoints for interpreting the susceptibility of *L. monocytogenes* to benzylpenicillin, ampicillin, meropenem, moxifloxacin, linezolid, erythromycin, and trimethoprim-sulfamethoxazole. Therefore, clindamycin susceptibility was interpreted following Moura et al. (2024), who established a breakpoint of 15 mm. Overall, 77.4% (48/62) of the strains exhibited inhibition zones <15 mm and were classified as resistant, whereas 22.6% (14/62) were susceptible (≥15 mm) (Figures 1, 2; Supplementary Table S2). Resistant strains were assigned to CCs 1, 3, 5, 6, 8, 9, 37, 87, 121, 155, and 475, while susceptible strains belonged to CCs 31 and 155. All strains within a CC showed uniform susceptibility profiles, except CC155. Within this CC, strains P8, P9, P11, R1, and R11 were susceptible, whereas strain C007 was resistant. A Kruskal–Wallis test revealed highly significant differences (*p* < 0.0001) in CC distribution between resistant and susceptible groups, indicating a lineage-linked resistance phenotype, and identifying CC31 and CC155 as consistently sensitive. Correlation analysis showed an almost perfect association (*ϕ* = 0.95) between clindamycin resistance and halo formation on CH-L medium. All non-halo-forming strains were susceptible and all resistant strains formed a halo, except C007. This association was confirmed by Fisher’s exact test (*p* < 0.0001), suggesting halo formation as a complementary phenotypic marker of clindamycin resistance.

*L. monocytogenes* is intrinsically resistant to several antibiotics, including cephalosporins, oxacillin, nalidixic acid, fosfomycin, sulfonamides, and clindamycin (CA-SFM and EUCAST, 2024). However, in this study, 77.4% of the strains analyzed were clindamycin resistant, which is consistent with previous reports describing a decrease in natural clindamycin resistance in *L. monocytogenes* (Sosnowski et al., 2019; Andriyanov et al., 2021; Moura et al., 2024; Castello et al., 2025). Clindamycin inhibits protein synthesis by binding to the 50S ribosomal subunit and blocking peptidyl transferase activity, thereby preventing peptide bond formation and elongation of the amino acid chain (Brodiazhenko et al., 2022; Wiśniewski et al., 2022). Natural resistance to clindamycin is mainly mediated by the chromosomal *vgaG* gene, encoding an ABC-F type ribosomal protection protein that interferes with antibiotic binding (Dar et al., 2016). Alterations in this gene or in its regulation may affect susceptibility and could explain the low concordance between phenotypic and genotypic clindamycin resistance reported (Moura et al., 2024). Additionally, plasmid-borne genes such as *lnuA*, *lnuB*, and *lnuG* may confer resistance (Chmielowska et al., 2021; Rodrigues et al., 2024), although this mechanism appears to be less frequent.

Likewise, the minimum inhibitory concentrations (MICs) of clindamycin were determined for 16 *L. monocytogenes* strains using the agar dilution method. According to the disk diffusion results, two strains were classified as resistant and 14 were susceptible. Resistant strains exhibited MICs of 2 μg/mL after 24 h of incubation at 35 °C, whereas susceptible strains showed lower values ranging from 0.25 to 0.5 μg/mL (Supplementary Table S2). These results are consistent with previous studies reporting MICs of 0.125–0.5 μg/mL for susceptible strains and 1–2 μg/mL for resistant strains (Brodiazhenko et al., 2022). Higher MIC values for resistant strains isolated from processing environments (1–32 μg/mL) have also been reported (Wiśniewski et al., 2022).

### 3.2 Genotypic characterization

#### 3.2.1 *In vitro* determination of clindamycin resistance genes

The presence of the resistance genes *lnuB*, *lnuG,* and *vgaG* was investigated by PCR in 62 *L. monocytogenes* strains, and the resulting sequences were compared with those of the reference strain ATCC BAA-679 to identify potential functional variations. According to the Microbial Browser for Identification of Genetic and Genomic Elements (MicroBIGG-E) database, these genes represent the most common clindamycin resistance determinants in *L. monocytogenes* (MicroBIGG-E, 2025). None of strains carried *lnuB* or *lnuG*, which encode lincosamide nucleotidyltransferases, whereas *vgaG*, encoding the ABC-F type ribosomal protection protein VgaG, was detected in all tested strains, including those susceptible to clindamycin (Figure 1; Supplementary Table S3). These findings are consistent with the literature, which reports high prevalence of *vgaG* and a low occurrence of the plasmid-borne *lnu* genes in *L. monocytogenes* (Wiśniewski et al., 2022; Parra-Flores et al., 2022; Moura et al., 2024).

Sequencing of the *vgaG* gene revealed 11 distinct alleles, each associated with a specific clonal complex (CC) (Figure 1; Supplementary Table S3). According to the Institut Pasteur BIGSdb-Lm, 385 different alleles have been submitted for this locus for *Listeria* spp. All strains within the same CC shared identical nucleotide sequences, except those from CC155. Clindamycin resistant strains exhibited 10 different alleles (1, 2, 3, 4, 5, 7, 10, 13, 15, and 33), whereas susceptible strains showed only two: a novel allele identified in CC155 and allele 28 in CC31 strains. The distribution of *vgaG* alleles differed significantly between resistant and susceptible strains (*p* < 0.0001), indicating that *vgaG* genotyping may discriminate between both phenotypes.

**Figure 3:**
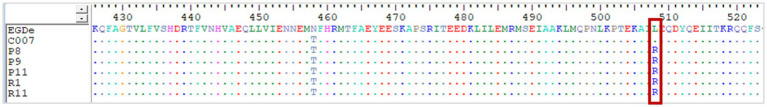
Amino acid polymorphisms of VgaG in *L. monocytogenes* CC155 strains. Amino acid sequences are aligned to the reference strain ATCC BAA-679 (EGD-e). Identical amino acids to the sequence in reference strain are presented as dots (.). Amino acids changes predicted to be deleterious by SIFT (*p* < 0.05) are presented in a red box.

The nucleotide sequences were translated into their corresponding amino acid sequences and variations in VgaG protein were evaluated. In susceptible CC155 strains (P8, P9, P11, R1, and R11), a leucine-to-arginine substitution at position 508 (Leu508Arg) was detected, whereas the resistant CC155 strain C007 showed a sequence identical to the reference strain (Figure 3). This mutation may explain the different behavior of CC155 strains toward clindamycin. In susceptible CC31 strains (A7, A13, P12, P18, P19, R4, R6, S1, C009), two non-synonymous substitutions were identified: a lysine-to-threonine change at position 152 (Lys152Thr) and an asparagine-to-threonine change at position 458 (Asn458Thr). Nevertheless, these mutations were also found in strains from other CCs that displaying phenotypic tolerance to clindamycin.

The VgaG protein consists of two nucleotide-binding domains, a resistance-specific domain, and a C-terminal extension (CTE), with clindamycin resistance mediated through ribosomal protection. When VgaG recognizes an antibiotic-blocked ribosome, it binds to it and triggers conformational changes that destabilize the antibiotic-binding site, thereby abolishing its activity. The Leu508Arg substitution is located within the CTE (amino acids 463–523), a region previously shown to be critical for resistance in homologous proteins (Crowe-McAuliffe et al., 2021). According to our results, this mutation may contribute to the loss of clindamycin resistance observed in CC155 strains, although functional studies are required to confirm its impact on protein function and its relationship with the observed susceptible phenotype.

The expression of *vgaG* is regulated through an attenuation mechanism controlled by a riboregulator located in the 5′ untranslated region (5′UTR), which adopts terminator or antiterminator conformations depending on antibiotic presence. Interaction of clindamycin with the ribosome promotes formation of the antiterminator structure, thereby allowing full transcription of the resistance gene. This mechanism has been demonstrated by mutagenesis, as disruption of the antiterminator region blocks transcription even in the presence of the antibiotic (Dar et al., 2016). Despite multiple sequence variations in VgaG, no specific changes were identified that fully explain clindamycin susceptibility in CC31 strains, nor were alterations detected in non-coding regulatory regions. These findings suggest that the low concordance between genotype and phenotype for clindamycin resistance, also reported in other studies (Moura et al., 2024), may be attributable to the complex regulation of this gene.

Although a substantial number of phenotypically susceptible *L. monocytogenes* strains have been registered, the presence of intrinsic clindamycin resistance genes, as well as genes coferring resistance to other antibiotics or disinfectants, remain a latent risk, due to the potential for inducible resistance, cross-resistance, and horizontal gene transfer under certain clinical or industrial conditions. Therefore, it is crucial to investigate this low phenotype–genotype concordance observed for clindamycin resistance, the mechanisms underlying this phenomenon and its relationship with virulence-associated traits.

#### 3.2.2 WGS analysis

**Figure 4:**
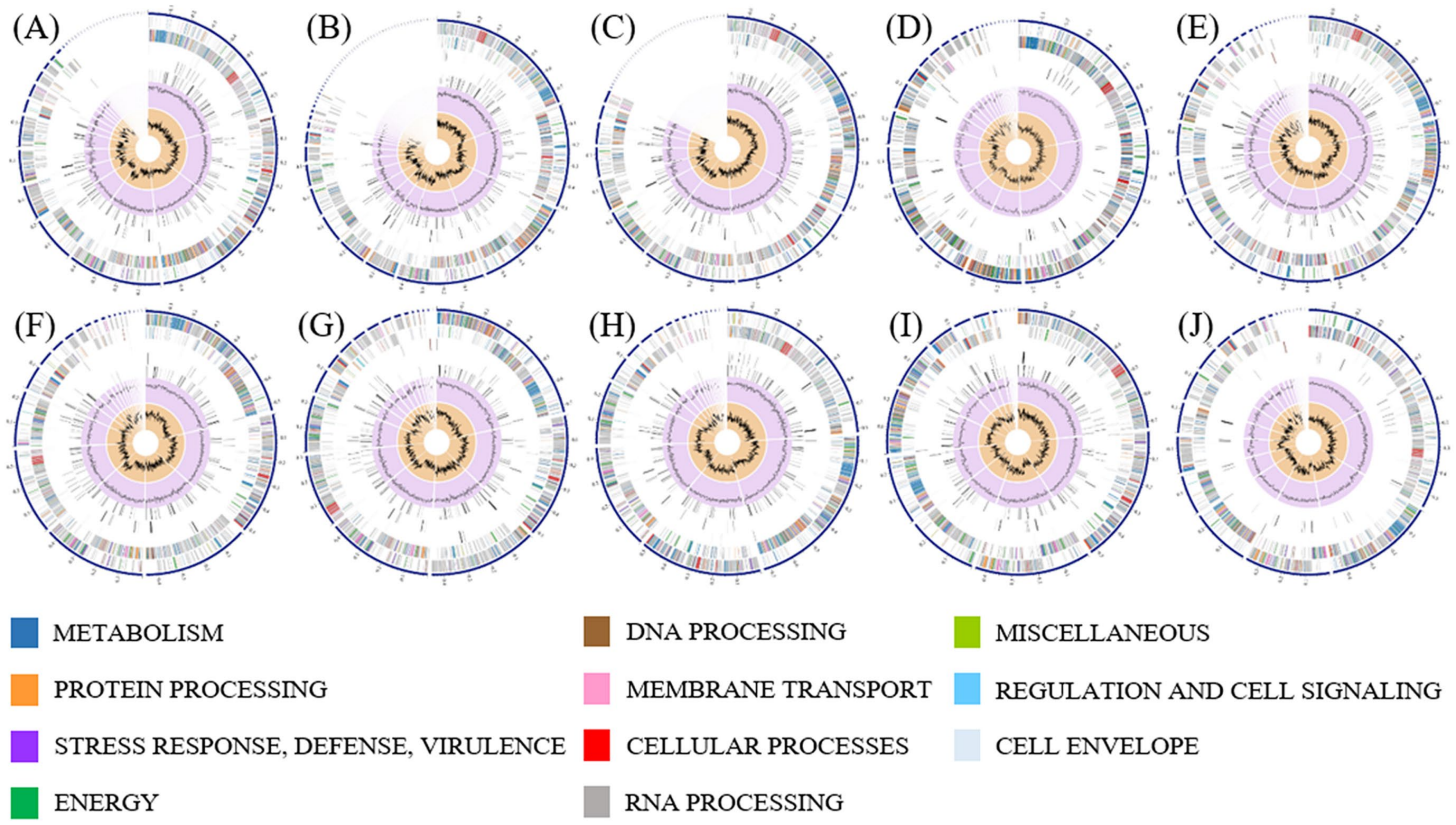
Circular graphical representation of the genome annotations of *L. monocytogenes* strains A7 **(A)**, A13 **(B)**, S1 **(C)**, C009 **(D)**, R4 **(E)**, P8 **(F)**, P9 **(G)**, R1 **(H)**, C007 **(I)**, and A001 **(J)**. From the outermost to the innermost rings, each plot displays: contigs, coding sequences (CDS) on the forward strand, CDS on the reverse strand, RNA genes, CDS with homology to known antimicrobial resistance genes, CDS with homology to known virulence factors, GC content, and GC skew. Colors used for CDS on the forward and reverse strands correspond to the subsystem to which each gene belongs.

Ten *L. monocytogenes* strains were selected for WGS analysis based on their clindamycin resistance profiles and their ability to produce halo on CH-L medium. Specifically, eight clindamycin susceptible strains were selected, belonging to CC31 (A7, A13, C009, R4, and S1) and CC155 (P8, P9, and R1), all of which did not form halo. In addition, two clindamycin resistant strains were included, one from CC155 (C007) and one from CC121 (A001), both of which formed halo on CH-L medium. The genomic characteristics obtained by WGS for the 10 *L. monocytogenes* strains are summarized in Table 2, with graphical genome representation shown in Figure 4 and Supplementary Figure S1. The average genome size was approximately 3.05 Mb. The G + C content was highly conserved (average 37.8%), consistent with values reported for the genus *Listeria* (Sallen et al., 1996), suggesting the absence of large horizontal acquisitions of foreign DNA. Genome size correlated strongly with the number of CDS (*r* = 0.99), with larger genomes (A13 and R4) containing around 3,180 CDS, and smaller genomes (C007) harboring 2,875 CDS. A notable inverse relationship was observed between rRNAs copy number and the abundance of repetitive regions. These patterns suggest two contrasting genomic configurations: one associated with higher translational efficiency and genome stability, and another reflecting greater genomic plasticity driving by repetitive sequences that may act as recombination hotspots (Klappenbach et al., 2000; Lupski and Weinstock, 1992). In contrast, tRNA gene numbers were conserved across strains, indicating similar translational capacity (Du et al., 2017). Approximately 19–21% of predicted proteins were annotated as hypothetical, highlighting the still large proportion of uncharacterized coding sequences. All strains exhibited high pathogenic potential, carrying between 90 and 95 virulence-associated genes, and showed a uniform antimicrobial resistance profile, with most strains harboring five resistance genes. The identification of virulence factors in *L. monocytogenes* using WGS is highly variable depending on the database used for the analysis, which may explain the large difference in virulence factors found in other studies (Gana et al., 2024).

**Table 2:** Genomic features of ten *L. monocytogenes* strains isolated from meat-processing industries.

Genome features	A7	A13	S1	C009	R4	P8	P9	R1	C007	A001
Genome size (pb)	3,088,362	3,112,968	2,997,617	3,081,984	3,104,616	3,118,937	3,119,287	3,119,052	2,893,551	3,075,509
No. of contigs	40	60	51	26	30	22	19	20	15	23
G + C content (%)	37.87	37.82	37.81	37.82	37.84	37.67	37.66	37.67	37.89	37.88
No. of CDS	3,137	3,182	3,040	3,152	3,188	3,173	3,178	3,174	2,875	3,100
No. of tRNA	56	56	59	57	57	58	51	58	58	57
No. of rRNA	7	8	9	3	3	3	3	3	3	3
Repeat regions	28	28	28	15	29	69	69	69	70	60
Hypothetical proteins	607	621	537	606	670	644	649	647	478	603
Proteins with functional assigments	2,530	2,561	2,053	2,546	2,518	2,529	2,529	2,527	2,397	2,497
Antimicrobial resistance^a^	5	5	5	6	6	5	5	5	5	5
Virulence factors^b^	90	91	92	91	90	95	95	95	95	90

CDS, Coding DNA Sequence.

a Databases: CARD (Comprehensive Antibiotic Resistance Database), Institute Pasteur Bacterial Isolate Genome Sequence Database and NCBI (National Center for Biotechnology Information).

b Database: VFDB (Virulence Factor Database).

**Figure 5:**
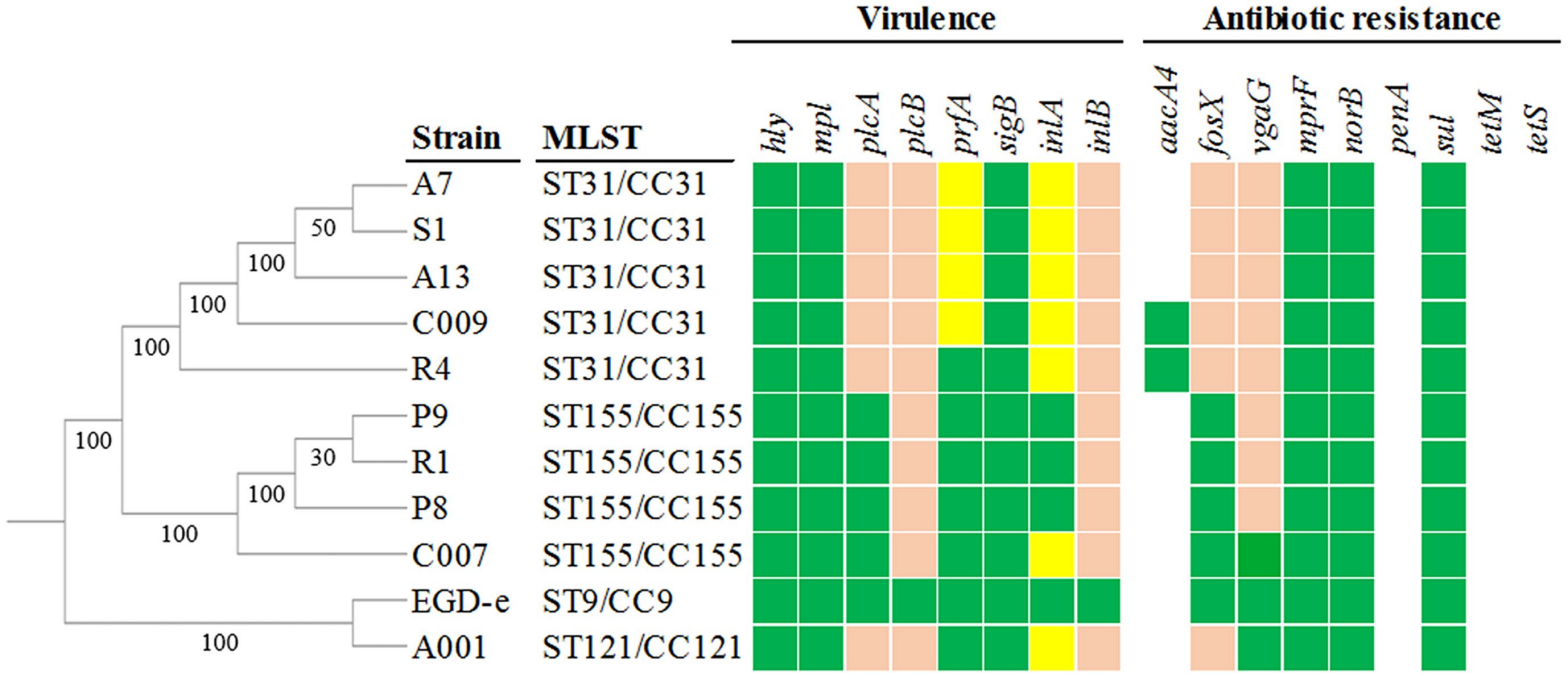
Phylogenetic relatedness and genetic determinants of *L. monocytogenes* strains associated with virulence and antibiotic resistance. Evolutionary distances are expressed as substitutions per site. Numbers shown at the nodes correspond to the confidence of each bifurcation, calculated by FastTree. The presence of known virulence factors and antibiotic resistance genes is indicated by green boxes in the heatmap. Yellow colored boxes represent the presence of truncated genes, and pink colored boxes are genes predicted to be deletereus by SIFT (*p* < 0.05).

Pangenome analysis using Roary identified 2,552 core genes (99–100% identity), while the accessory genome comprised 1,037 shell genes (15–95% identity) and 420 cloud genes (0–15% identity). These cloud genes were strain-specific, with 348 encoding hypothetical proteins. Synteny analysis performed with Mauve (Supplementary Figure S1) revealed a conserved core genome structure but extensive chromosomal rearrangements, including inversions and translocations, indicating local genomic plasticity within a globally stable architecture. Phylogenetic analysis showed minimal divergence among strains within the same ST and modest variation between CCs, consistent with all strains belonging to lineage II of *L. monocytogenes,* regardless of their origin (Figure 5; Supplementary Figure S1).

Overall, these results demonstrate that *L. monocytogenes* strains from meat-processing environments maintain stable genome sizes, while exhibiting considerable structural variability. This variability likely reflects strain-specific evolution, accumulation of mobile elements, and adaptive processes occurring in food-production settings. Such genomic flexibility may influence the expression of stress-response or virulence traits, and underscores the importance of whole-genome approaches for understanding the evolutionary dynamics of *L. monocytogenes* in industrial environments.

#### 3.2.3 *In silico* analysis of antimicrobial resistance genes

The genomes of 10 *L. monocytogenes* strains were screened for antimicrobial resistance genes. Intrinsic resistance determinants to fosfomycin (*fosX* – *lmo1702*), quinolones (*norB* – *lmo2818*), cationic peptides (*mprF* – *lmo1695*), sulfonamides (*sul – lmo0224*), and lincosamides (*vgaG – lmo0919*) were detected in all strains (Figure 5). Protein sequences encoded by these genes were analyzed and compared with those of the reference strain ATCC BAA-679 to identify functionally relevant variations. No amino acid polymorphisms were observed in MprF and Sul, whereas sequence variations were detected in FosX, NorB and VgaG. In CC31 strains, several FosX substitutions (Lys43Gln, Glu101Gln, Arg105Ser, Ser106Trp) were predicted by SIFT to affect functionality (*p* < 0.05). The CC121 strain also harbored two deleterious FosX substitutions. CC155 displayed multiple substitutions in NorB, none predicted to impact the protein functionality. Regarding the VgaG protein, the WGS analysis confirmed the results reported in Section 3.2.1.

Concerning acquired resistance, only the aminoglycoside resistance gene *aacA4* was detected, and only in two CC31 strains (R4 and C009), both of which remained phenotypically susceptible to gentamycin according to the EUCAST disk diffusion testing (data not shown). These findings aling with previous studies reporting widespread intrinsic resistance genes in *L. monocytogenes* but rare acquisition of additional resistance determinants (Moura et al., 2024; Mafuna et al., 2021; Hong et al., 2025; Parra-Flores et al., 2022).

Plasmids and mobile genetic elements potentially associated with resistance genes were also investigated. Strain A001 (CC121), harbored the pLM5578 plasmid, a replicon commonly associated with CC121 isolates, which contains cadmium resistance genes *cadA* and *cadC*, and has been link to bacterial adaptation, disinfectant tolerance, and long-term persistence in the food-processing environments (Gilmour et al., 2010; Mafuna et al., 2021). This strain also harbored the transposon Tn*6188*, which carries the *qacH* gene and is associated with resistance to quaternary ammonium compounds, a feature considered to contribute to the high prevalence of CC121 isolates in the food industry (Pérez-Baltar et al., 2021). All CC31 strains and three CC155 strains (P8, P9 and R1) carried the pLM33 plasmid, previously associated with tolerance to multiple environmental stressors, such as low pH, high salt concentrations, oxidative conditions, and elevated temperatures (Liu et al., 2025). In contrast, strain C007 did not harbor any plasmid or mobile genetic element. Furthermore, strains A7 and A13 (CC31) carried the *bcrABC* cassette, a determinant known to confer resistance to benzalkonium chloride (BAC) and often plasmid-borne in *L. monocytogenes* (Dutta et al., 2013; Elhanafi et al., 2010).

Overall, *L. monocytogenes* genomes are enriched in diverse mobile genetic elements that support their ability to colonize and persist in a wide range of ecological niches by providing additional adaptive traits (Chmielowska et al., 2021). Although none of the identified plasmids carried antibiotic resistance genes, several efflux pump systems, such as *bcrABC* and *qacH* were detected, both of which have been associated with resistance to multiple antimicrobials and may also confer cross protection against certain antibiotics (Kim et al., 2018; López-Alonso et al., 2020). Identifying strains that exhibit tolerance to biocides commonly used in food-processing environments is particularly important, as exposure to sub-inhibitory concentrations of disinfectants can select for phenotypes with enhanced efflux activity, thereby promoting cross resistance between disinfectants and antibiotics (Bland et al., 2022; Douarre et al., 2022; Kode et al., 2021). Nevertheless, the precise molecular mechanisms underlying these cross-resistance phenomena remain incompletely understood, and further research is required to clarify their functional significance and impact on food safety.

#### 3.2.4 *In silico* analysis of virulence factors

Virulence-associated genes was also screened in the genomes of the 10 selected *L. monocytogenes* strains. The pathogenicity island LIPI-1, including *hly* (listeriolysin O), *actA* (actin polymerization protein), *plcA* and *plcB* (phospholipases C), *mpl* (metalloprotease), and its transcriptional regulator, *prfA*, were detected in all strains (Figure 5). Likewise, PrfA-regulated genes located outside LIPI-1, such as *inlA* and *inlB* (internalins A and B), which are essential for cell invasion, were detected in all strains. The stress response regulator *sigB*, which plays an important role in the adaptation to environmental stresses and regulates transcription of the *prfA* virulence gene cluster, was also detected in all strains. In contrast, the pathogenicity islands LIPI-2 and LIPI-3 were absent, consistent with their low prevalence among food-related *L. monocytogenes* isolates (Gana et al., 2024; Hong et al., 2025; Hurley et al., 2019; Parra-Flores et al., 2022). These genes were examined to identify sequence variations and to explore a potential relationship between clindamycin susceptibility and virulence of *L. monocytogenes* strains.

Three distinct *prfA* alleles were identified, with notable differences among CC31 strains. While strain R4 displayed a *prfA* sequence identical to those found in CC155 strains and in the reference strain ATCC BAA-679, strains A7, A13, S1, and C009 exhibited a 7 nucleotide insertion (CAGGAGT) at position 521 of the *prfA* coding sequence. This alteration caused a frameshift that generated a premature stop codon (PMSC) at amino acid 185, resulting in a truncated PrfA protein. Such alterations at this position have been previously reported in CC31 strains and are associated with loss of PrfA-regulated virulence gene expression and with a non-virulent phenotype better adapted to a non-pathogenic extracellular lifestyle (Velge et al., 2007; Maury et al., 2017). Neither the remaining strain CC31 (R4) nor the strains belonging to CC155 and CC121 exhibited polymorphisms affecting the *prfA* sequence, indicating that PrfA functionality is likely preserved in those clonal complexes.

**Figure 6:**
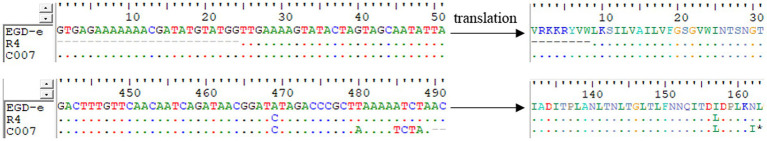
The *inlA* mutations identified in two *L. monocytogenes* strains (R4 and C007 belonging to clonal complex 31 and 155, respectively). Sequences are aligned to the reference strain ATCC BAA-679 (EGD-e). Identical nucleotides/amino acids to the sequence in reference strain are presented as dots (.). Deletions or non-sense mutations are indicated by a hyphen (−). Premature stop codon is indicated by an asterisk (*).

InlA, encoded by the *inlA* gene, is a key virulence determinant responsible for mediating host cell adhesion through binding to E-cadherin in epithelial cells (Koopmans et al., 2023) and plays an essential role in intestinal translocation during early stages of infection (Lecuit et al., 2001). This virulence factor can harbor a wide variety of PMSCs that lead to truncated proteins associated with attenuated pathogenicity (Li et al., 2024). In this study, six distinct *inlA* alleles were detected, of which only three lacked PMSCs. PMSC6 was detected in the CC121 strain A001 and corresponded to a cytosine-to-thymine substitution at nucleotide 1,474 (amino acid 492). This mutation has been specifically associated with *L. monocytogenes* CC121 strains and with attenuated virulence traits (Ragon et al., 2008; Pérez-Baltar et al., 2021). Likewise, PMSC5 was detected in CC31 strains A7, A13, S1, and C009, caused by a cytosine-to-thymine substitution at nucleotide 565 (amino acid 189), and has previously linked to hypovirulent phenotypes (Van Stelten et al., 2010). The CC31 strain R4 carried a novel *inlA* allele characterized by a deletion of the first 24 nucleotides upstream of the start codon (Figure 6), potentially affecting secretion and surface localization of InlA (Schubert et al., 2002). Such an alteration may compromise the ability of InlA to bind its E-cadherin receptor, impairing its functionality (Bierne and Cossart, 2007; Bonazzi et al., 2009; Hamon et al., 2006). Additionally, the strain C007 (CC155) carried a thymine deletion at nucleotide 480, producing a frameshift and a truncated InlA protein of 162 amino acids (Figure 6). This mutation was not detected in any of the other CC155 strains analyzed (P8, P9, and R1), and to our knowledge, this PMSC represents a novel *inlA* allele not previously recorded in BIGSdb-Lm database or reported in the literature. Parra-Flores et al. (2022) and Ji et al. (2023) also identified undescribed PMSCs in *L. monocytogenes* food-associated isolates, suggesting that the diversity of *inlA* truncations is likely underestimated, making it necessary to update the literature. PMSCs in *inlA* occur more frequently in food-related isolates than in clinical ones (Ferreira da Silva et al., 2017) and, as they are associated with a loss of InlA functionality, may result in a markedly reduced ability of *L. monocytogenes* to invade epithelial cells, leading to non-virulent or strongly attenuated phenotypes. Recent large-scale genomic analyses have reinforced that *inlA* integrity is one of the primary predictors of virulence potential (Russini et al., 2025).

InlB, which is less restrictive than InlA, promotes pathogen entry into various cell types, including hepatocytes, epithelial cells, and endothelial cells (Bierne and Cossart, 2002). Three alleles of the *inlB* gene were identified, each associated with one of the three CCs analyzed. All alleles harbored mutations relative to the reference strain, resulting in amino acid substitutions in InlB that may potentially reduce protein functionality (*p* < 0.05 according to SIFT). Strains isolated from food-processing environments frequently carry truncated or non-functional variants of this protein, in contrast to clinical strains. In this context, Hurley et al. (2019) reported internal deletions in the *inlB* sequence in 11% of isolates obtained from food-processing plants using WGS.

Although allelic variants of *hly* and *mpl* were detected, none were predicted to produce non-functional proteins. Listeriolysin O (LLO), encoded by *hly*, is a pore-forming toxin which allows *L. monocytogenes* entry into the cytoplasm of the host cell by lysis of the phagocytic vacuole (Vázquez-Boland et al., 2001). LLO consists of 529 amino acids and is produced as a precursor protein of 554 amino acids that undergoes signal peptide cleavage. The *mpl* gene encodes a 510-amino acid metalloprotease, which together with PlcA and PlcB, contributes to vacuole lysis and supports LLO activity (Camejo et al., 2011). In the present study, although functional, a previously undescribed *mpl* allele, not listed in the Institut Pasteur database, was identified in strain A001. The *actA* gene, which mediates intracellular motility and cell-to-cell spread (Cheng et al., 2018), encodes a 639-amino acid protein. Here, CC31 strains as well as strain A001 (CC121) harbored 604-amino acid ActA variant, a modification that may attenuate their virulence.

The alternative factor SigB, encoded by the *sigB* gene, enhances *L. monocytogenes* survival under diverse environmental conditions, including osmotic, acid and bile stress, and acts as a transcriptional regulator of stress-response genes. Additionally, SigB modulates the expression of several virulence genes such as *prfA, inlA,* and *inlB*, playing a key role in pathogenity (De las Heras et al., 2011; Kazmierczak et al., 2006). In this study, two allelic variants of the *sigB* gene were identified, with strain A001 carrying the only distinct sequence. Despite this variation, all alleles encoded functional proteins.

The *plcA* gene exhibited several amino acid substitutions in CC31 strains (Asn4Ile, Gln7Arg and Tyr19Cys), which were predicted to be deleterious by SIFT (*p* < 0.05). The Gln7Arg substitution was also detected in strain A001 (CC121). In contrast, a Tyr17Val substitution was observed in CC155 strains, but this alteration was predicted to be tolerated by SIFT. These substitutions are located in the N-terminal region of the signal peptide, where modifications may disrupt interactions necessary for proper secretion. In addition, all strains showed amino acid substitutions in the PlcB sequence relative to the ATCC BAA-679 strain and these changes were predicted by SIFT to be non-tolerated. *L. monocytogenes* synthesizes two phospholipases C, phosphatidylinositol-specific phospholipase A (PlcA, encoded by *plcA*) and broad-spectrum phospholipase B (PlcB, encoded by *plcB*), both of which play a key role in vacuole lysis during the infection cycle (Reissbrodt, 2004; Koopmans et al., 2023) and alterations in them can reduce virulence by impairing the bacterium ability to escape from the host cell vacuole. Phenotypically, CC31 and CC155 strains (except C007) failed to form halo in CH-L medium. Whereas this observation was consistent with the genomic findings in CC31 strains, no *plcA* mutations were identified in CC155 strains that could impair phosphatidylinositol hydrolysis on CH-L medium and thus prevent the formation of the characteristic halo. No relevant genomic differences were detected between strain C007 and the other CC155 strains that could account for the observed phenotypic discrepancy, suggesting differences in the regulatory pathways controlling *plcA* expression. All CC155 strains analyzed, except C007, were isolated from the same food-processing plant, whereas strain C007 was recovered from a different facility. Distinct environmental conditions may have influenced regulatory pathways controlling *plcA* expression or secretion. Alternatively, metabolic differences affecting local environmental conditions, such as pH, may impair PlcA activity and halo formation.

In this study, *L. monocytogenes* strains exhibiting clindamycin susceptibility also harbored multiple truncated virulence genes, resulting in attenuated virulence and a distinctive phenotype on CH-L medium. Consistent with these findings, previous studies have reported a low prevalence of antibiotic-resistant *L. monocytogenes* phenotypes in food and food-processing environments worldwide (Li et al., 2022; Parra-Flores et al., 2022). Correlation analysis revealed a moderate positive association between clindamycin resistance or susceptibility and certain virulence genes, including *prfA, inlA, inlB,* and *plcB* (*ϕ* ≈ 0.50) as well as *actA* (*ϕ* ≈ 0.30). Although statistical significance could not be established due to the limited sample size (N = 11), these consistent trends suggest a biologically meaningful link between reduced virulence and clindamycin susceptibility. Overall, these results provide a foundation for future studies and highlight patterns that should be validated using larger datasets. The low frequency of antibiotic resistance in food-related environments, where strains tend to adapt to a non-pathogenic lifestyle, reflects the absence of the strong selective pressures typical of clinical settings. Under these conditions, clindamycin susceptibility may act as an indirect indicator of reduced pathogenic potential. Conversely, previous studies have shown that *L. monocytogenes* strains resistant to certain antibiotics under environmental conditions may become susceptible once the pathogenic cycle is activated. Scortti et al. (2018) demonstrated that a fosfomycin-resistant strain became susceptible upon activation of virulence genes due to epistatic gene regulation between *fosX*, *prfA* and *hpt.* Together, these observations underscore the need for elucidate the mechanisms underlying antibiotic resistance in *L. monocytogenes*, with the ultimate goal of improving treatment strategies in the event of infection.

## 4 Conclusion

This study reveals limited concordance between phenotypic and genotypic profiles of clindamycin resistance in *L. monocytogenes* from meat and meat-processing environments. Although all strains carried the intrinsic resistance gene *vgaG*, specific mutations, together with lineage dependent genomic variation, were associated with clindamycin susceptibility. Acquired antibiotic resistance genes were rare, and the resistome was largely limited to intrinsic determinants. Clindamycin susceptible strains frequently harbored truncated or functionally impaired virulence genes, particularly *prfA, inlA, inlB, actA,* and *plcA*, consistent with attenuated virulence and loss of PI-PLC activity on CH-L medium. These findings suggest that clindamycin susceptibility in food-associated *L. monocytogenes* may be linked to reduced pathogenic potential, yet they also underscore the complexity of predicting antimicrobial phenotypes from genotypes in this species. Given the influence of environmental factors, biocide exposure, and regulatory interactions on the modulation of resistance expression, continued integration genomic and phenotypic surveillance throughout the food-processing chain is crucial for identifying emerging variants and informing control strategies that protect food safety and public health.

## Data Availability

The datasets presented in this study can be found in online repositories. The names of the repository/repositories and accession number(s) can be found in the article/Supplementary material.
