# Supplementary material for: Clindamycin susceptibility and virulence characterization of Listeria monocytogenes strains isolated from meat and meat-processing environments
Source: Front Microbiol. 2026 May 21;17:1833569. doi: 10.3389/fmicb.2026.1833569 (PMC13233710; doi:10.3389/fmicb.2026.1833569)
Supplement: Supplementary file 1 [file Data_Sheet_1.PDF]

Table S1. *L. monocytogenes* strains isolated from meat-processing industries included in this study.

	Strain	Serogroup	Sequence Type	Clonal Complex	Plant	Sample	Collection period
1	A1	IIb	ST5	CC5	A (broiler abattoir)	Carcasse/Environment	2004-2005
2	A3	IIa	ST121	CC121	A (broiler abattoir)	Environment	2004-2005
3	A7	IIa	ST31	CC31	A (broiler abattoir)	Environment	2004-2005
4	A10	IIa	ST121	CC121	A (broiler abattoir)	Environment	2004-2005
5	A13	IIa	ST31	CC31	A (broiler abattoir)	Environment	2004-2005
6	P1	IVb	ST710	CC1	A (poultry processing plant)	Finished product	2005
7	P2	IIc	ST9	CC9	A (poultry processing plant)	Finished product	2005
8	P3	IIc	ST9	CC9	A (poultry processing plant)	Finished product	2005
9	P5	IIb	ST5	CC5	A (poultry processing plant)	Finished product	2005
10	P8	IIa	ST3055	CC155	A (poultry processing plant)	Finished product	2005
11	P9	IIa	ST155	CC155	A (poultry processing plant)	Finished product	2005
12	P11	IIa	ST155	CC155	A (poultry processing plant)	Finished product	2005
13	P12	IIa	ST31	CC31	A (poultry processing plant)	Finished product	2005
14	P15	IIb	ST517	CC9	A (poultry processing plant)	Finished product	2005
15	P16	IIb	ST423	CC3	A (poultry processing plant)	Finished product	2005
16	P17	IIb	ST3	CC3	A (poultry processing plant)	Finished product	2005
17	P18	IIa	ST31	CC31	A (poultry processing plant)	Finished product	2005
18	P19	IIa	ST31	CC31	A (poultry processing plant)	Finished product	2005
19	P22	IIa	ST121	CC121	A (poultry processing plant)	Finished product	2005
20	P24	IIb	ST5	CC5	A (poultry processing plant)	Finished product	2005
21	R1	IIa	ST155	CC155	A (retail)	Raw food	2005
22	R2	IIb	ST423	CC3	A (retail)	Raw food	2005

23	R4	IIa	ST31	CC31	A (retail)	Raw food	2005
24	R6	IIa	ST31	CC31	A (retail)	Raw food	2005
25	R8	IIc	ST9	CC9	A (retail)	Raw food	2005
26	R11	IIa	ST155	CC155	A (retail)	Raw food	2005
27	EE41	IVb	ST2404	CC6	C (pork processing plant)	Environment	2014
28	S1	IIa	ST31	CC31	B (pork processing plant)	Raw product/Environment/Finished product	2005-2008
29	S4-1	IIb	ST87	CC87	B (pork processing plant)	Raw product/Environment	2005-2008
30	S4-2	IIb	ST87	CC87	B (pork processing plant)	Raw product/Environment/Finished product	2005-2008
31	S5	IIc	ST9	CC9	B (pork processing plant)	Raw product/Environment	2005-2008
32	S7-1	IVb	ST422	CC6	B (pork processing plant)	Raw product	2005-2008
33	S7-2	IVb	ST422	CC6	B (pork processing plant)	Raw product	2005-2008
34	S7-3	IVb	ST422	CC6	B (pork processing plant)	Raw product	2005-2008
35	S7-4	IVb	ST422	CC6	B (pork processing plant)	Raw product	2005-2008
36	S8	IIc	ST9	CC9	B (pork processing plant)	Raw product/Environment	2005-2008
37	S9	IVb	ST710	CC1	B (pork processing plant)	Raw product	2005-2008
38	S12-1	IIc	ST9	CC9	B (pork processing plant)	Raw product/Environment/Finished product	2005-2008
39	S13	IIb	ST5	CC5	B (pork processing plant)	Raw product	2005-2008
40	S17-1	IIb	ST423	CC3	B (pork processing plant)	Raw product/Environment	2005-2008
41	S18	IIb	ST87	CC87	B (pork processing plant)	Raw product	2005-2008
42	S21	IIc	ST9	CC9	B (pork processing plant)	Environment	2005-2008
43	A001	IIa	ST121	CC121	C (pork processing plant)	Environment	2018
44	A004	IIb	ST3	CC3	C (pork processing plant)	Environment	2018
45	A007	IIc	ST9	CC9	C (pork processing plant)	Environment	2018
46	A009	IIa	ST121	CC121	C (pork processing plant)	Environment	2018
47	B001	IIc	ST9	CC9	D (pork processing plant)	Environment	2014-2015

48	B002	IIb	ST87	CC87	D (pork processing plant)	Environment	2014-2015
49	B007	IIa	ST121	CC121	D (pork processing plant)	Environment	2014-2015
50	B012	IIb	ST87	CC87	D (pork processing plant)	Environment	2014-2015
51	B021	IVb	ST1	CC1	D (pork processing plant)	Environment	2014-2015
52	C001	IIa	ST236	CC121	E (pork processing plant)	Environment	2014-2019
53	C002	IIa	ST121	CC121	E (pork processing plant)	Environment	2014-2019
54	C003	IIa	ST504	CC475	E (pork processing plant)	Environment	2014-2019
55	C004	IIa	ST8	CC8	E (pork processing plant)	Environment	2014-2019
56	C007	IIa	ST155	CC155	E (pork processing plant)	Environment	2014-2019
57	C008	IIa	ST236	CC121	E (pork processing plant)	Environment	2014-2019
58	C009	IIa	ST31	CC31	E (pork processing plant)	Environment	2014-2019
59	C010	IIa	ST8	CC8	E (pork processing plant)	Environment	2014-2019
60	C021	IIa	ST121	CC121	E (pork processing plant)	Environment	2014-2019
61	C022	IIc	ST9	CC9	E (pork processing plant)	Environment	2014-2019
62	C025	IIa	ST37	CC37	E (pork processing plant)	Environment	2014-2019

Table S2. Phenotypic characterization of *L. monocytogenes* strains isolated from meat-processing industries. “++”: halo size similar to the reference strain *L. monocytogenes* ATCC BAA-679 (EGD-e); “–”: absence of halo; nd: not determined; DA: clindamycin (2 µg); R: resistant; S: susceptible.

	Strain	Halo CH-L	Halo DA (mm)	MIC DA (µg/mL)	Susceptibility DA
Control	EGD-e	++	10	2	R
Control	ATCC 29213	nd	26	nd	S
1	A1	++	12	nd	R
2	A3	++	12	nd	R
3	A7	–	23	0,25	S
4	A10	++	11	nd	R
5	A13	–	22	0,25	S
6	P1	++	11	nd	R
7	P2	++	10	nd	R
8	P3	++	11	nd	R
9	P5	++	11	nd	R
10	P8	–	20	0,25	S
11	P9	–	20	0,25	S
12	P11	–	20	0,25	S
13	P12	–	21	0,25	S
14	P15	++	10	nd	R
15	P16	++	10	nd	R
16	P17	++	12	nd	R
17	P18	–	22	0,5	S
18	P19	–	21	0,5	S
19	P22	++	12	nd	R
20	P24	++	12	nd	R
21	R1	–	19	0,25	S
22	R2	++	11	nd	R
23	R4	–	20	0,5	S
24	R6	–	21	0,25	S
25	R8	++	10	nd	R
26	R11	–	20	0.25	S
27	EE41	++	13	nd	R
28	S1	–	28	0,25	S
29	S4-1	++	14	nd	R
30	S4-2	++	13	nd	R
31	S5	++	13	nd	R
32	S7-1	++	14	nd	R
33	S7-2	++	13	nd	R
34	S7-3	++	13	nd	R

35	S7-4	++	13	nd	R
36	S8	++	13	nd	R
37	S9	++	10	nd	R
38	S12-1	++	13	nd	R
39	S13	++	10	nd	R
40	S17-1	++	10	nd	R
41	S18	++	10	nd	R
42	S21	++	14	nd	R
43	A001	++	10	2	R
44	A004	++	12	nd	R
45	A007	++	14	nd	R
46	A009	++	9	nd	R
47	B001	++	14	nd	R
48	B002	++	14	nd	R
49	B007	++	10	nd	R
50	B012	++	13	nd	R
51	B021	++	10	nd	R
52	C001	++	11	nd	R
53	C002	++	10	nd	R
54	C003	++	10	nd	R
55	C004	++	11	nd	R
56	C007	++	12	2	R
57	C008	++	10	nd	R
58	C009	−	27	0,25	S
59	C010	++	11	nd	R
60	C021	++	10	nd	R
61	C022	++	13	nd	R
62	C025	++	14	nd	R

Table S3. Presence of lincosamides resistance genes among *L. monocytogenes* strains isolated from meat-processing industries. DA: clindamycin; “+”: presence of the gene; “–”: absence of the gene.

		DA resistance genes			
	Strain	*lnuB*	*lnuG*	*vgaG*	*vgaG allele*
Control	EGD-e	–	–	+	1
1	A1	–	–	+	4
2	A3	–	–	+	10
3	A7	–	–	+	28
4	A10	–	–	+	10
5	A13	–	–	+	28
6	P1	–	–	+	2
7	P2	–	–	+	1
8	P3	–	–	+	1
9	P5	–	–	+	4
10	P8	–	–	+	13
11	P9	–	–	+	13
12	P11	–	–	+	13
13	P12	–	–	+	28
14	P15	–	–	+	1
15	P16	–	–	+	3
16	P17	–	–	+	3
17	P18	–	–	+	28
18	P19	–	–	+	28
19	P22	–	–	+	10
20	P24	–	–	+	4
21	R1	–	–	+	13
22	R2	–	–	+	3
23	R4	–	–	+	28
24	R6	–	–	+	28
25	R8	–	–	+	1
26	R11	–	–	+	1
27	EE41	–	–	+	5
28	S1	–	–	+	28
29	S4-1	–	–	+	15
30	S4-2	–	–	+	15
31	S5	–	–	+	1
32	S7-1	–	–	+	5
33	S7-2	–	–	+	5

34	S7-3	–	–	+	5
35	S7-4	–	–	+	5
36	S8	–	–	+	1
37	S9	–	–	+	2
38	S12-1	–	–	+	1
39	S13	–	–	+	4
40	S17-1	–	–	+	3
41	S18	–	–	+	15
42	S21	–	–	+	1
43	A001	–	–	+	10
44	A004	–	–	+	3
45	A007	–	–	+	1
46	A009	–	–	+	10
47	B001	–	–	+	1
48	B002	–	–	+	15
49	B007	–	–	+	10
50	B012	–	–	+	15
51	B021	–	–	+	2
52	C001	–	–	+	10
53	C002	–	–	+	10
54	C003	–	–	+	10
55	C004	–	–	+	7
56	C007	–	–	+	13
57	C008	–	–	+	10
58	C009	–	–	+	28
59	C010	–	–	+	7
60	C021	–	–	+	10
61	C022	–	–	+	1
62	C025	–	–	+	33
