# Supplementary material for: Clindamycin susceptibility and virulence characterization of Listeria monocytogenes strains isolated from meat and meat-processing environments
Source: Front Microbiol. 2026 May 21;17:1833569. doi: 10.3389/fmicb.2026.1833569 (PMC13233710; doi:10.3389/fmicb.2026.1833569)
Supplement: Supplementary file 2 [file Data_Sheet_2.PDF]

Figure S1. Comparison of the complete genomes of 11 strains of *L. monocytogenes*, including isolates obtained from meat processing environments and the reference strain EGD-e, generated using the BV-BRC Genome Alignment Service and visualized with the PATRIC Mauve Viewer. Horizontal bars represent full genome length (kb). Colored syntenic blocks indicate conserved genomic regions, where identical colors and positions reflect shared gene order. Crossing lines between blocks denote genomic rearrangements (translocation or inversion), whereas vertical alignment of blocks indicates conserved synteny. For each strain, blocks above central axis represent regions in the forward orientation (5' to 3'), and blocks below represent regions inverted with respect to the reference genome. White gaps imply genetic differences or indels and the absence of connecting lines indicates loss of the corresponding genomic segment in the compared strain.
