# Supplementary figures and images for: Clindamycin susceptibility and virulence characterization of Listeria monocytogenes strains isolated from meat and meat-processing environments
Source: Front Microbiol. 2026 May 21;17:1833569. doi: 10.3389/fmicb.2026.1833569 (PMC13233710; doi:10.3389/fmicb.2026.1833569)

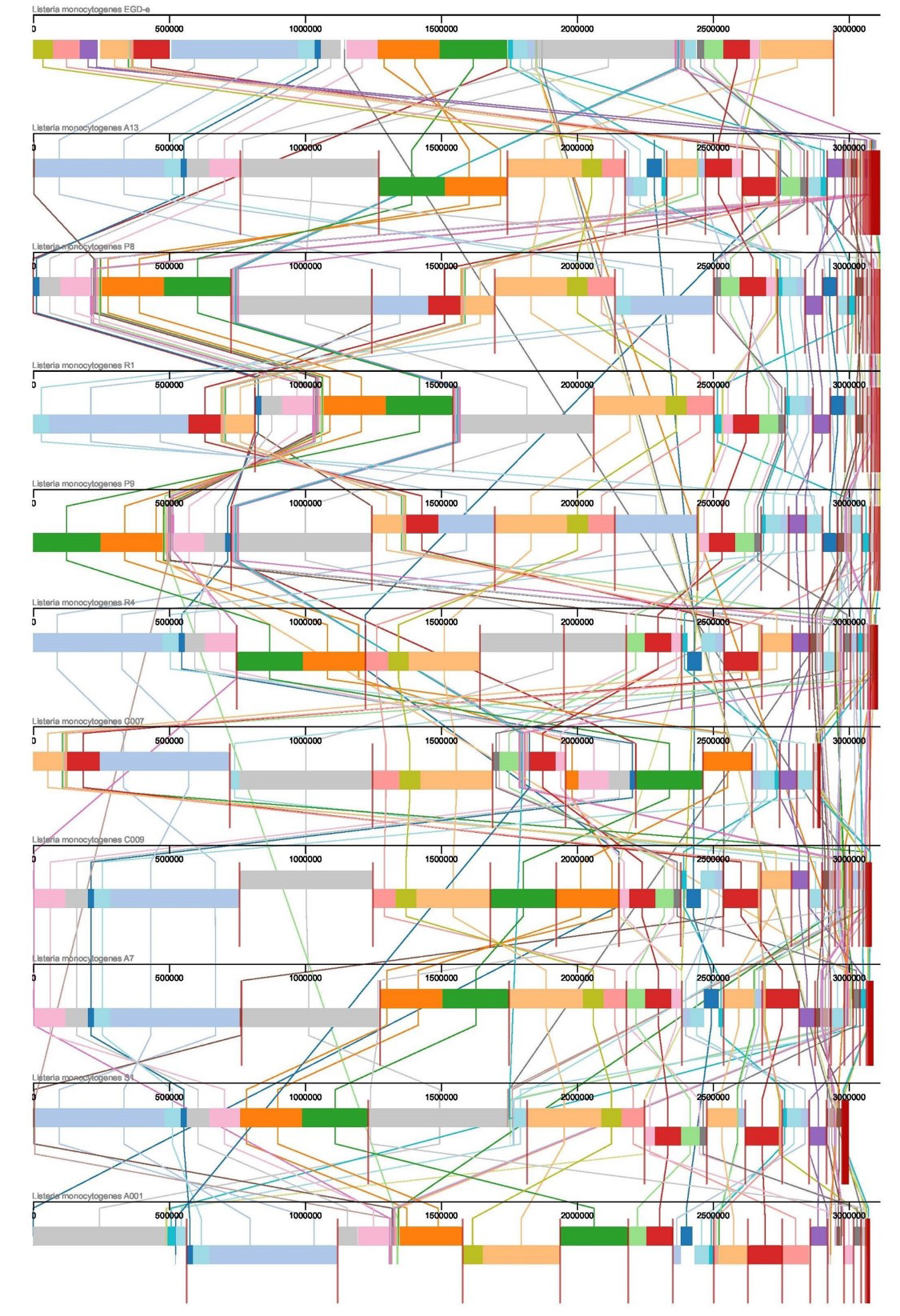

Supplement: Supplementary file 3 [file Image_1.TIF]
